# Development and validation of multiparameter prognostic nomogram combining neutrophil-to-lymphocyte ratio, tumor burden, and nutritional status for predicting postoperative outcome in elderly gastric cancer patients

**DOI:** 10.3389/fonc.2025.1620443

**Published:** 2025-09-03

**Authors:** Dongyuan Liu, Yan Jiang, Min Gao

**Affiliations:** ^1^ Department of General Surgery, No.971 Hospital of People’s Liberation Army Navy, Qingdao, China; ^2^ Department of Medical Care, No.971 Hospital of People’s Liberation Army Navy, Qingdao, China; ^3^ Department of Geriatrics, Shandong Provincial Hospital Affiliated to Shandong First Medical University, Jinan, China

**Keywords:** elderly gastric cancer, the ratio of neutrophil to lymphocytes, clinical outcomes, nomogram prediction model, validation

## Abstract

**Objective:**

This study aims to construct and verify Nomogram prediction model for clinical outcomes of elderly patients with gastric cancer after surgery, which is based on multiple factors such as peripheral blood neutrophil to lymphocyte ratio (NLR), tumor burden and nutritional status.

**Methods:**

A total of 189 elderly patients with gastric cancer who received surgical treatment in our hospital from January 2019 to December 2024 were included in the study. They were divided into a training set (n=132) and a validation set (n=57) according to the ratio of 7:3. The data including age, gender, body mass index (BMI), smoking history, drinking history, comorbidities, tumor location, size, histological type, differentiation degree, TNM stage, lymph node metastasis, and preoperative NLR value were collected. After single-factor and multi-factor analysis, the risk factors influencing the postoperative clinical outcome were screened, and the Nomogram model was constructed, evaluated and validated. The decision curve analysis was used to evaluate the clinical value of the model.

**Results:**

There were 46 cases (34.85%) with adverse clinical outcomes in the training set and 20 cases (35.09%) with adverse clinical outcomes in the validation set. Multivariate Logistic regression analysis showed that NLR, BMI, tumor size, lymph node metastasis, carcinoembryonic antigen (CEA), and age were the independent influencing factors for adverse clinical outcomes of elderly patients after gastric cancer surgery (all *P* < 0.05). The calibration degree and prediction performance of the nomogram model in the training set and the validation set were good. The C-index index was 0.806 and 0.879, respectively. The calibration curves showed that the average absolute errors of the predicted values and the true values were 0.172 and 0.110, respectively. The Hosmer-Lemeshow test results were χ^2^ = 16.669, *P*=0.034 and χ^2^ = 5.653, *P*=0.686, respectively. AUC values were 0.802(95% *CI*: 0.708-0.895) and 0.888(95% *CI*: 0.757-1.000), respectively, with sensitivity and specificity of 0.889, 0.650, and 0.900 and 0.654, respectively.

**Conclusion:**

The NLR-based Nomogram model is useful for predicting the postoperative clinical outcomes of elderly patients with gastric cancer, but it needs to be verified by further large-sample multi-center research.

## 1 Introduction

Gastric cancer is a common malignant tumor that seriously threatens human health, and its incidence and mortality remain high in China (1). With the aggravation of aging population, the number of elderly patients with gastric cancer increases. The decline of body function of this group, often accompanied by a variety of chronic diseases, makes the postoperative clinical outcome complex and the prognosis is different. Therefore, accurate prediction of postoperative clinical outcomes in elderly patients with gastric cancer is essential for formulating personalized treatment options and improving the prognosis (2). The neutrophil-to-lymphocyte ratio (NLR) in peripheral blood, as a key indicator reflecting systemic inflammatory and immune status, has emerged as a crucial biomarker for cancer prognosis (3). Elevated NLR levels demonstrate enhanced systemic inflammation and immune suppression, thereby mirroring tumor cell proliferation, invasion, and metastatic potential. For elderly gastric cancer patients with inherently compromised immune function, NLR serves as a sensitive marker for evaluating postoperative anti-tumor immunity and recovery capacity (4). These properties justified its inclusion as a study parameter. However, NLR alone cannot comprehensively predict postoperative outcomes due to the multifactorial nature of clinical results influenced by additional variables. Carcinoembryonic antigen (CEA) is a common tumor marker and has great significance in the diagnosis and treatment of gastric cancer (5). When gastric cancer occurs, tumor cells secrete CEA, resulting in an increase in its level in the blood. High CEA levels are often associated with high tumor invasiveness and poor prognosis (6). In addition, factors such as age, tumor TNM stage, lymph node metastasis, tumor differentiation degree and comorbidities can also significantly affect the prognosis of patients with gastric cancer. Elderly patients have poor tolerance to surgery and treatment. Late tumor staging, multiple lymph node metastases, low degree of differentiation and the combination of chronic diseases will increase the risk of surgery and affect rehabilitation. At present, there are few studies on the prediction model of clinical outcomes after surgery for gastric cancer in the elderly constructed by combining NLR and CEA with the above various factors. This study aims to fill this gap, build a multi-factor Nomogram prediction model, provide an accurate prediction tool for clinical practice, guide the treatment and management of elderly patients with gastric cancer, and improve the survival rate and quality of life of patients.

## 2 Materials and methods

### 2.1 Subjects

A total of 189 elderly patients with gastric cancer who received surgical treatment in our hospital from January 2019 to December 2024 were selected as the research subjects. Inclusion criteria: (1) Age ≥60 years old; (2) Gastric cancer is confirmed by pathology; (3) Receiving radical surgery; (4) Patients did not receive chemoradiotherapy, targeted therapy or immunotherapy before operation; (5) Complete clinical data, including preoperative NLR test results of peripheral blood, surgical records, postoperative pathological report, and follow-up data; (6) Patients or families signed informed consent form. Exclusion criteria: (1) Those complicated with other malignant tumors; (2) Severe dysfunction of heart, liver, lung, kidney and other vital organs is present; (3) Active stage combined with hematological system diseases, autoimmune diseases or infectious diseases; (4) The medical records are incomplete or the interviewee is missing. Patients were divided into a training set and a validation set in a 7:3 ratio using a completely randomized approach. The study was approved by the Ethics Committee of The Shandong Provincial Hospital Affiliated to Shandong First Medical University (No. 2024SDPH-11043), and informed consent was obtained from all patients. This study was conducted in accordance with the Declaration of Helsinki.

### 2.2 Data collection

General clinical data of the patients were collected, including age, gender, height, weight, body mass index (BMI), smoking history, alcohol consumption history, and concomitant underlying diseases (such as hypertension, diabetes, and coronary heart disease). Record the tumor-related information of the patient in detail, such as tumor site (fundus of the stomach, stomach body, and gastric antrum), tumor size, histological type (adenocarcinoma, squamous cell carcinoma), differentiation degree (high/medium differentiation, and low differentiation), TNM staging of the tumor [according to the 8th Edition of AJCC Gastric Cancer Staging System, which is widely used for gastric cancer staging and focuses on tumor invasion depth, lymph node metastasis, and distant metastasis (7)], lymph node metastasis, carcinoembryonic antigen (CEA), etc. The fasting peripheral venous blood of the patient was collected preoperatively, and the neutrophil count and lymphocyte count were detected by an automatic blood cell analyzer, and NLR (NLR= neutrophil count/lymphocyte count) was calculated. This study implemented a standardized data collection protocol ensuring complete data availability for all 189 included patients, with a 0% missing rate across all analyzed variables. The following quality control measures were enforced:(1) Baseline clinical data were extracted directly from electronic medical records with cross-validation by two independent researchers. (2) Tumor characteristics were exclusively derived from definitive postoperative pathological reports validated by two pathologists. (3) Preoperative laboratory markers—mandatory tests for gastric cancer surgery patients—had fully traceable and complete records. (4) Follow-up was conducted by dedicated personnel through outpatient reviews and telephone interviews, achieving 100% follow-up completion without attrition.

### 2.3 Postoperative follow-up

Patients were followed up regularly after operation, including outpatient re-examination and telephone follow-up. Follow-up was calculated from the date of surgery until the patient’s death, loss of follow-up, or December 31, 2024. The main observation endpoint was the total survival of the patients, and the survival time and cause of death of the patients were recorded. Reference to relevant clinical guidelines: The NCCN Guidelines for Gastric Cancer (2024 Version) and ESMO Clinical Practice Guidelines for Gastric Cancer recommend using 3-year survival as an important indicator for evaluating postoperative prognosis and treatment response in elderly patients, especially for risk stratification and follow-up strategy formulation. Criteria for clinical outcome: The poor clinical outcome was the survival time of patients < 3 years after operation, and the good clinical outcome was ≥3 years.

### 2.4 Statistical analysis

SPSS26.0 and R 4.5.3 were used for statistical analysis. Counting data are expressed by the number of cases (percentage), and the comparison between groups is made by χ^2^ test; The measurement data conform to the normal distribution with S, the comparison between groups is tested by independent sample T, and the non-normal distribution with M (Q1, Q3) and Mann-Whitney U test. Multivariate Logistic regression analysis screened the influencing factors, and P < 0.05 was statistically significant. The variable selection for multivariable analysis followed a two-step approach: Initially, variables associated with postoperative clinical outcomes were screened using univariable logistic regression. Subsequently, multivariable logistic regression analysis was performed employing stepwise backward elimination. Throughout this process, multicollinearity among variables was assessed using the variance inflation factor (VIF); VIF < 10 indicated no significant multicollinearity. Variables retaining statistical significance (*P* < 0.05) after adjusting for other factors and demonstrating no multicollinearity were identified as independent influencing factors. In R language, the nomogram model is constructed with “rms” package, and the model is verified internally by Bootstrap method, and the calibration curve between the predicted results and the actual results is drawn, and the Concordance index (C-index) of the model is calculated. Hosmer-Lemeshow test is used to evaluate the goodness of fit of the predicted model. Clinical application value of decision curve analysis (DCA) model. All statistical tests were two-sided, with a significance level set at α = 0.05.

## 3 Results

### 3.1 Comparison of baseline characteristics data between the training set and the validation set

A total of 189 pancreatic cancer patients who were randomly divided into a training set (n = 132) and a validation set (n = 57) were selected. There was no significant difference in general clinical characteristics such as age, gender, BMI and most laboratory indicators between the two groups (all *P* > 0.05) (**Table 1**).

**Table 1:** Comparison of baseline characteristics s of patients between training set and validation set.

Indicators		Training set (n = 132)	Validation set (n = 57)	Statistical values	*P*-value
Age (years)		65.32 ± 5.12	66.11 ± 4.82	0.991	0.323
Gender	Male	78 (59.09)	35 (61.40)	0.089	0.766
	Female	54 (40.91)	22 (38.60)		
BMI (kg/m²)		23.20 ± 3.01	23.51 ± 2.83	0.661	0.509
Smoking history	Yes	52 (39.39)	23 (40.35)	0.015	0.902
	No	80 (60.61)	34 (59.65)		
Drinking history	Yes	45 (34.09)	20 (35.09)	0.018	0.895
	No	87 (65.91)	37 (64.91)		
Hypertension	Yes	48 (36.36)	21 (36.84)	0.004	0.950
	No	84 (63.64)	36 (63.16)		
Diabetes	Yes	50 (37.88)	22 (38.60)	0.009	0.926
	No	82 (62.12)	35 (61.40)		
NLR		2.63 ± 1.01	2.54 ± 0.92	0.577	0.565
CEA (ng/ml)		4.85 ± 1.24	4.77 ± 1.25	0.406	0.685
Tumor TNM staging	Stages i-ii	74 (56.06)	36 (63.16)	0.824	0.364
	Stage iii-iv	58 (43.94)	21 (36.84)		
Lymph node metastasis	Yes	61 (46.21)	26 (45.61)	0.006	0.940
	No	71 (53.79)	31 (54.39)		
Tumor site	Fundus of stomach	22 (16.67)	9 (15.79)		
	Gastric body	45 (34.09)	18 (31.58)		
	Gastric antrum	65 (49.24)	30 (52.63)		
Tumor size (cm)		4.31 ± 1.53	4.35 ± 1.41	0.169	0.866
Histological type	Glandular cancer	115 (87.12)	50 (87.72)	0.013	0.910
	Other	17 (12.88)	7 (12.28)		
Degree of differentiation	High/medium differentiation	85 (64.39)	33 (57.89)	0.717	0.397
	Poorly differentiated	47 (35.61)	24 (24.11)		
Distant metastasis	Yes	63 (47.73)	28 (49.12)	0.031	0.860
	No	69 (52.27)	29 (50.88)		
Tumor infiltration degree	Early stage	72 (54.55)	32 (56.14)	0.041	0.840
	Period of expansion	60 (45.45)	25 (43.86)		

### 3.2 Univariate analysis of influencing factors for clinical outcomes of postoperative gastric cancer in the elderly

There were 46 cases (34.85%) with adverse clinical outcomes in the training set and 20 cases (35.09%) with adverse clinical outcomes in the Validation set. The results of univariate analysis showed that there were significant differences between patients with adverse clinical outcomes and patients with good clinical outcomes in NLR, BMI, tumor size, lymph node metastasis, CEA, age and other indicators (P < 0.05), and there was no co-linearity among the covariates (the tolerance of each variable in the regression model > 0.1, VIF < 10, and condition index < 30, and the proportion of variances of multiple covariates without the same feature value > 50%) (**Table 2**).

**Table 2:** Univariate analysis of risk factors for clinical outcomes of postoperative gastric cancer in the elderly.

Indicators		Good clinical outcome (n = 86)	Poor clinical outcome (n = 46)	statistical values	*P*-value
Age (years)		64.23 ± 4.81	66.55 ± 5.54	2.503	0.013
gender	Male	50 (58.14)	28 (60.87)	0.092	0.761
	Female	36 (41.86)	18 (39.13)		
BMI (kg/m²)		22.17 ± 2.93	23.42 ± 3.16	2.272	0.025
Smoking history	Yes	32 (37.21)	20 (43.48)	0.493	0.483
	No	54 (62.79)	26 (56.52)		
Drinking history	Yes	29 (33.72)	16 (34.78)	0.015	0.903
	No	57 (66.28)	30 (65.22)		
hypertension	Yes	22 (25.58)	26 (56.52)	12.398	0.001
	No	64 (74.42)	20 (43.48)		
diabetes	Yes	38 (44.19)	12 (26.09)	0.454	0.501
	No	48 (55.81)	34 (73.91)		
NLR		2.34 ± 0.85	3.15 ± 1.17	4.794	0.001
CEA (ng/mL)		4.57 ± 1.50	5.32 ± 1.00	3.046	0.003
Tumor TNM staging	Stages i-ii	56 (65.12)	18 (39.13)	8.216	0.004
	Stage iii-iv	30 (34.88)	28 (60.87)		
Lymph node metastasis	Yes	34 (39.53)	27 (58.70)	4.427	0.035
	No	52 (60.47)	19 (41.30)		
Tumor site	Fundus of stomach	14 (16.28)	8 (17.39)	0.076	0.963
	Gastric body	30 (34.88)	15 (32.61)		
	Gastric antrum	42 (48.84)	23 (50.00)		
Tumor size (cm)		4.09 ± 1.42	4.73 ± 1.61	2.231	0.027
Histological type	Glandular cancer	75 (87.21)	40 (86.96)	0.002	0.967
	Other	11 (12.79)	6 (13.04)		
Degree of differentiation	High/medium differentiation	61 (70.93)	24 (52.17)	4.598	0.032
	Poorly differentiated	25 (29.07)	22 (47.83)		
Distant metastasis	Yes	35 (40.70)	28 (60.87)	4.888	0.027
	No	51 (59.30)	18 (39.13)		
Tumor infiltration degree	Early stage	53 (61.63)	19 (41.30)	4.993	0.026
	Period of expansion	33 (28.37)	27 (58.70)		

### 3.3 Multivariate logistic regression analysis

The postoperative clinical outcome (good clinical outcome =0, adverse clinical outcome =1) was used as the dependent variable, and the factor P<0.05 in the univariate analysis was used as the covariate for multivariate Logistic regression analysis (variable assignment table shown in **Table 3**). The results showed that NLR, BMI, tumor size, lymph node metastasis, CEA, and age were the independent risk factors for adverse clinical outcomes of elderly patients with gastric cancer after operation (*p* < 0.05) (**Table 4**).

**Table 3:** variable assignment method.

Variable	Meaning	Evaluation
X1	NLR	continuous variable
X2	BMI	continuous variable
X3	Tumor size	continuous variable
X4	Lymph node metastasis	Have =0, none =1
X5	CEA	continuous variable
X6	Age	continuous variable
Y	Postoperative clinical outcomes	Good clinical outcome =0, Poor clinical outcome =1

**Table 4:** Multivariate analysis of adverse clinical outcome in training set.

Indicators	*B*	Standard error	*Wald*	*P*-value	*OR*	95% CI
NLR	0.882	0.260	11.464	0.001	2.415	1.450-4.022
BMI	0.174	0.073	5.666	0.017	1.190	1.031-1.374
Tumor size	0.512	0.174	8.677	0.003	1.669	1.187-2.347
Lymph node metastasis	-1.215	0.471	6.649	0.010	0.297	0.118-0.747
CEA	0.481	0.177	7.363	0.007	1.618	1.143-2.291
Age	0.092	0.046	4.046	0.044	1.097	1.002-1.200
Constant	-17.064	4.062	17.650	0.001	0.001	

### 3.4 Construction of nomogram prediction model

**Figure 1:**
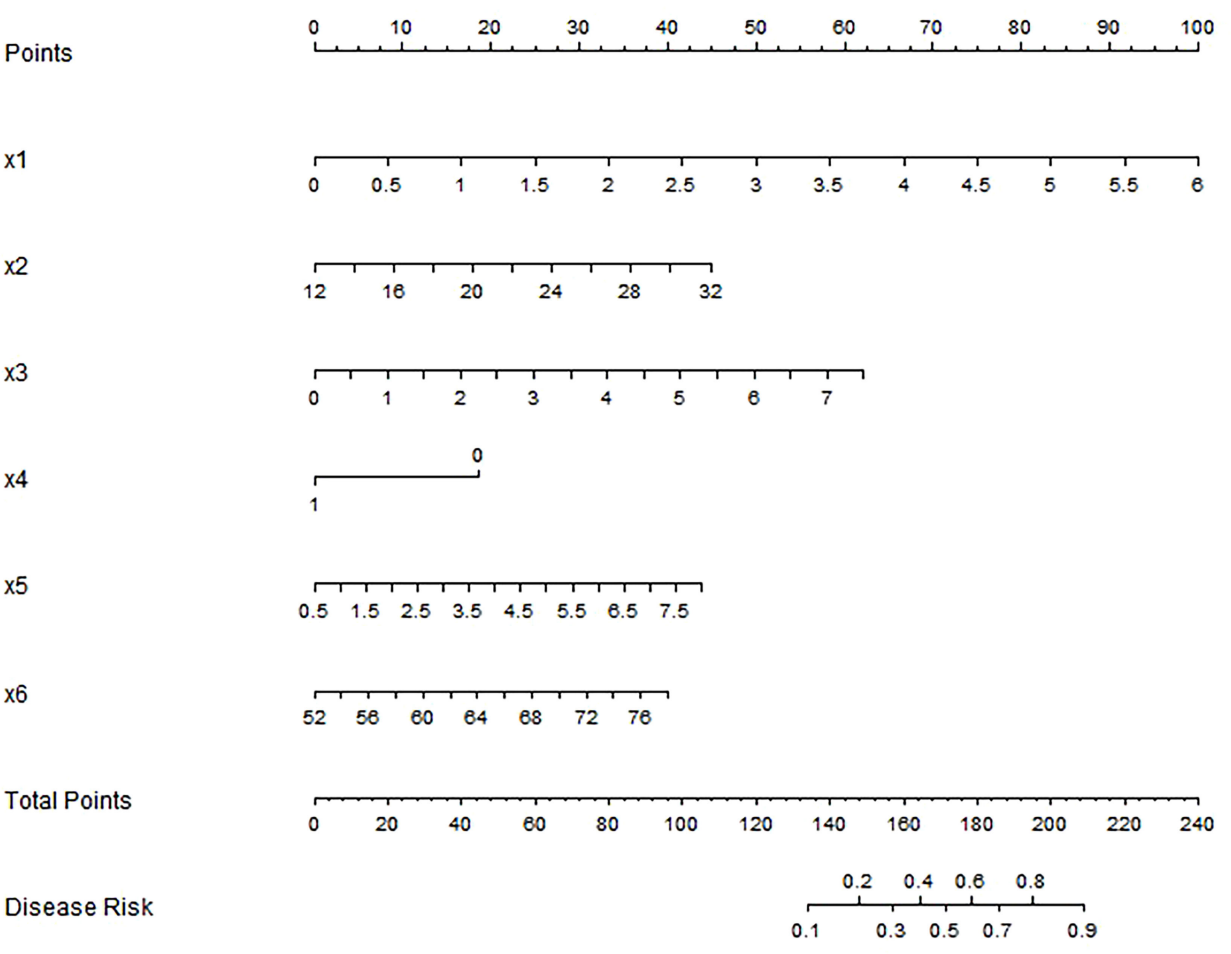
Nomogram prediction model for clinical outcome of postoperative gastric cancer in the elderly. Note: x1: NLR, x2:BMI, X3: Tumor size, X4: Lymph node metastasis; x5:CEA, X6: Age.

Based on the independent risk factors determined by multivariate Logistic regression analysis, we constructed the Nomogram prediction model for clinical outcomes of elderly patients with gastric cancer after surgery. Each independent influencing factor in the model was scored, and the total score for predicting adverse clinical outcomes after surgery was calculated, expressed as the probability of predicting poor clinical outcomes (**Figure 1**). A higher total score indicates a greater predicted risk of poor clinical outcomes.

### 3.5 Assessment and validation of nomogram prediction model for clinical outcomes of elderly patients with gastric cancer after surgery

**Figure 2:**
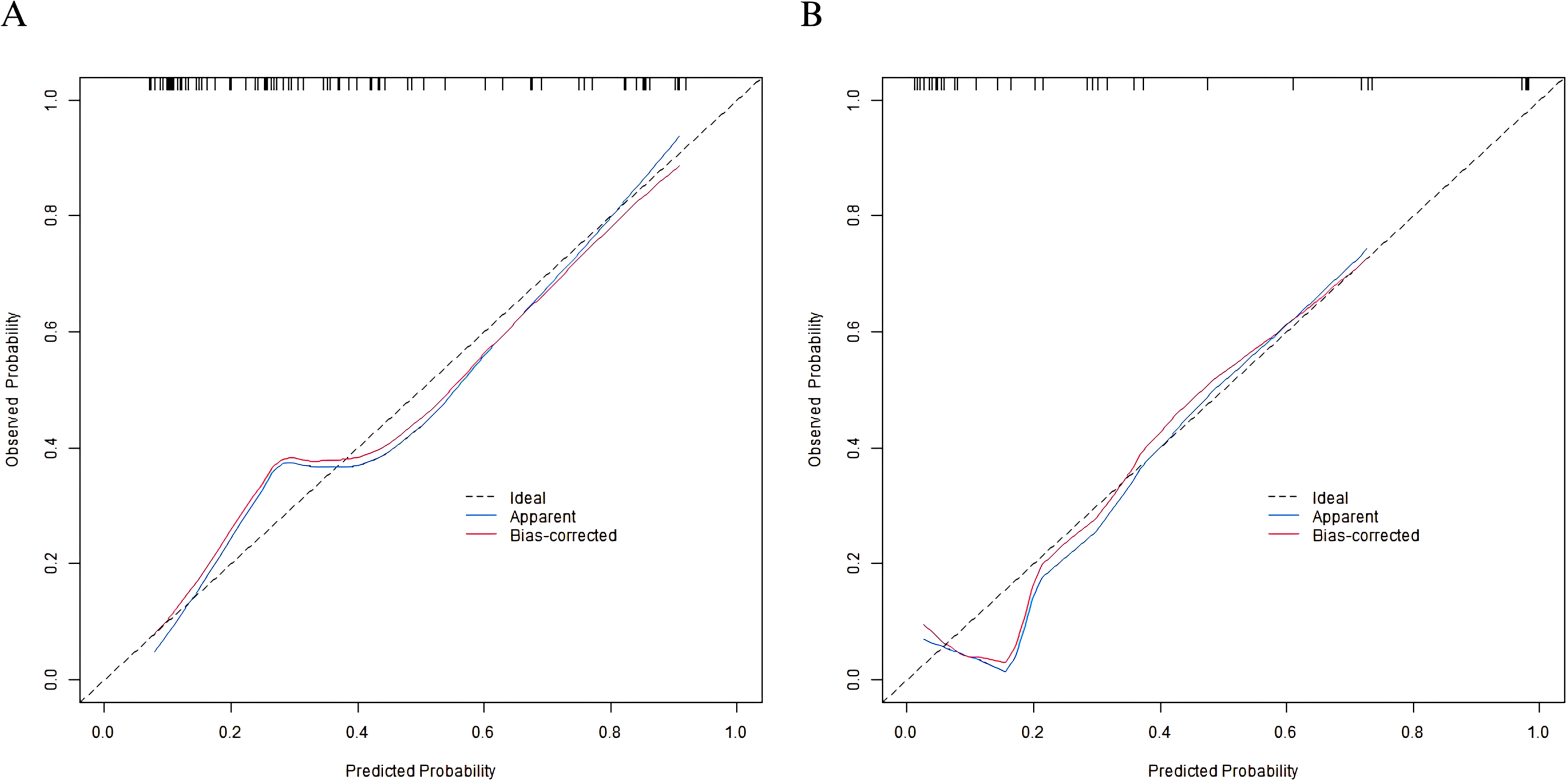
Calibration curves in the training set **(A)** and the validation set **(B)**.

**Figure 3:**
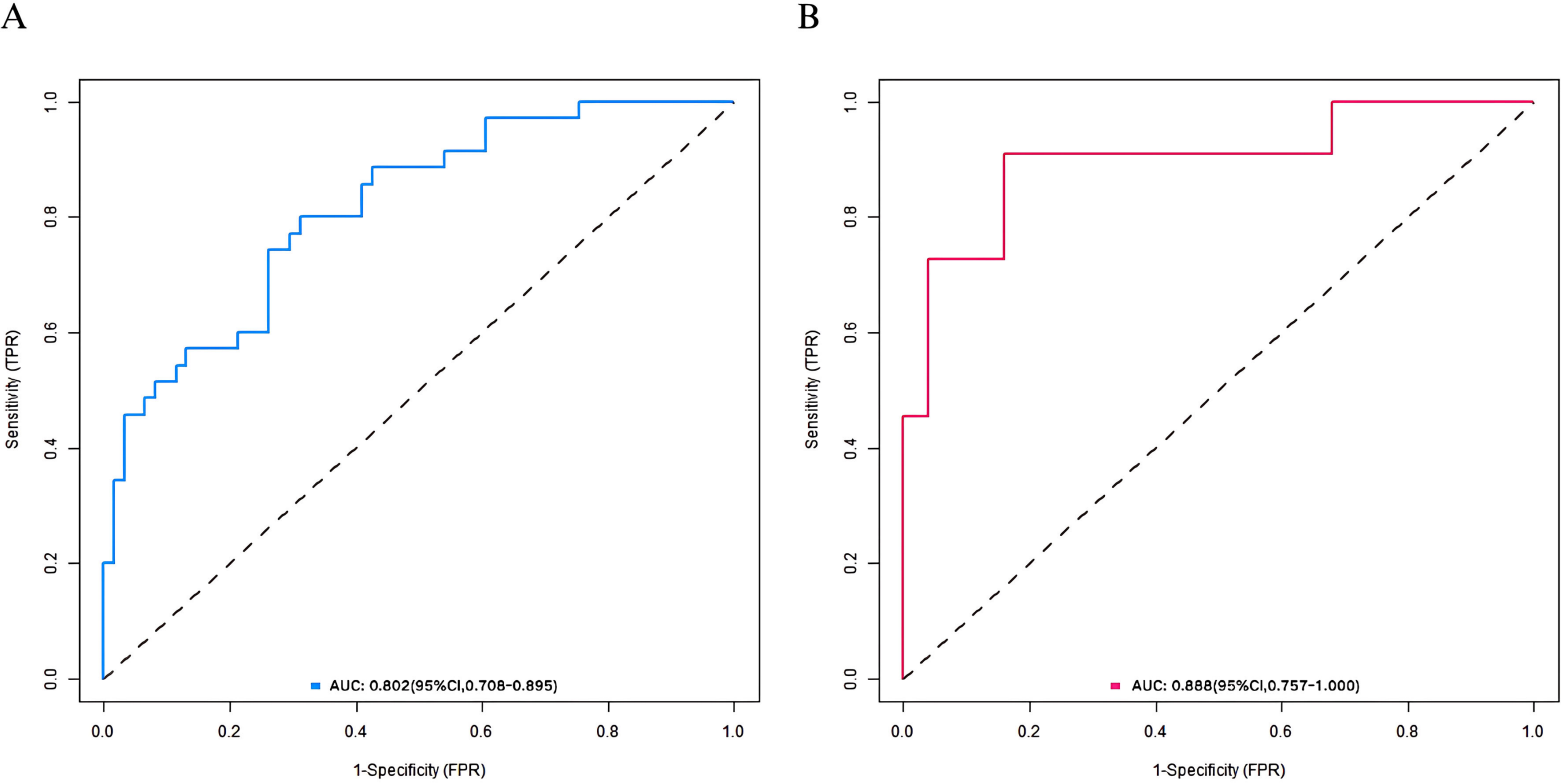
ROC curves in the training set **(A)** and the validation set **(B)**.

In the training set and Validation set, the C-index values of the nomogram model were 0.806 and 0.879, respectively. The calibration curves showed that the mean absolute errors of predicted values and actual values were 0.172 and 0.110, respectively. The Hosmer-Lemeshow test results were χ2 = 16.669, P=0.034 and χ2 = 5.653, P=0.686, respectively. The ROC curve was shown in the effective group and the ineffective group. The AUC of the nomogram model for predicting the postoperative clinical outcome of elderly gastric cancer patients was 0.802 (95% CI: 0.708–0.895) and 0.888 (95% CI: 0.757–1.000), respectively. The sensitivity and specificity were 0.889, 0.650, 0.900 and 0.654, respectively. The calibration curve and ROC curve are shown in **Figures 2**, **3**, respectively.

### 3.6 Decision-making curve analysis of nomogram prediction model for clinical outcomes of elderly patients with gastric cancer after surgery

**Figure 4:**
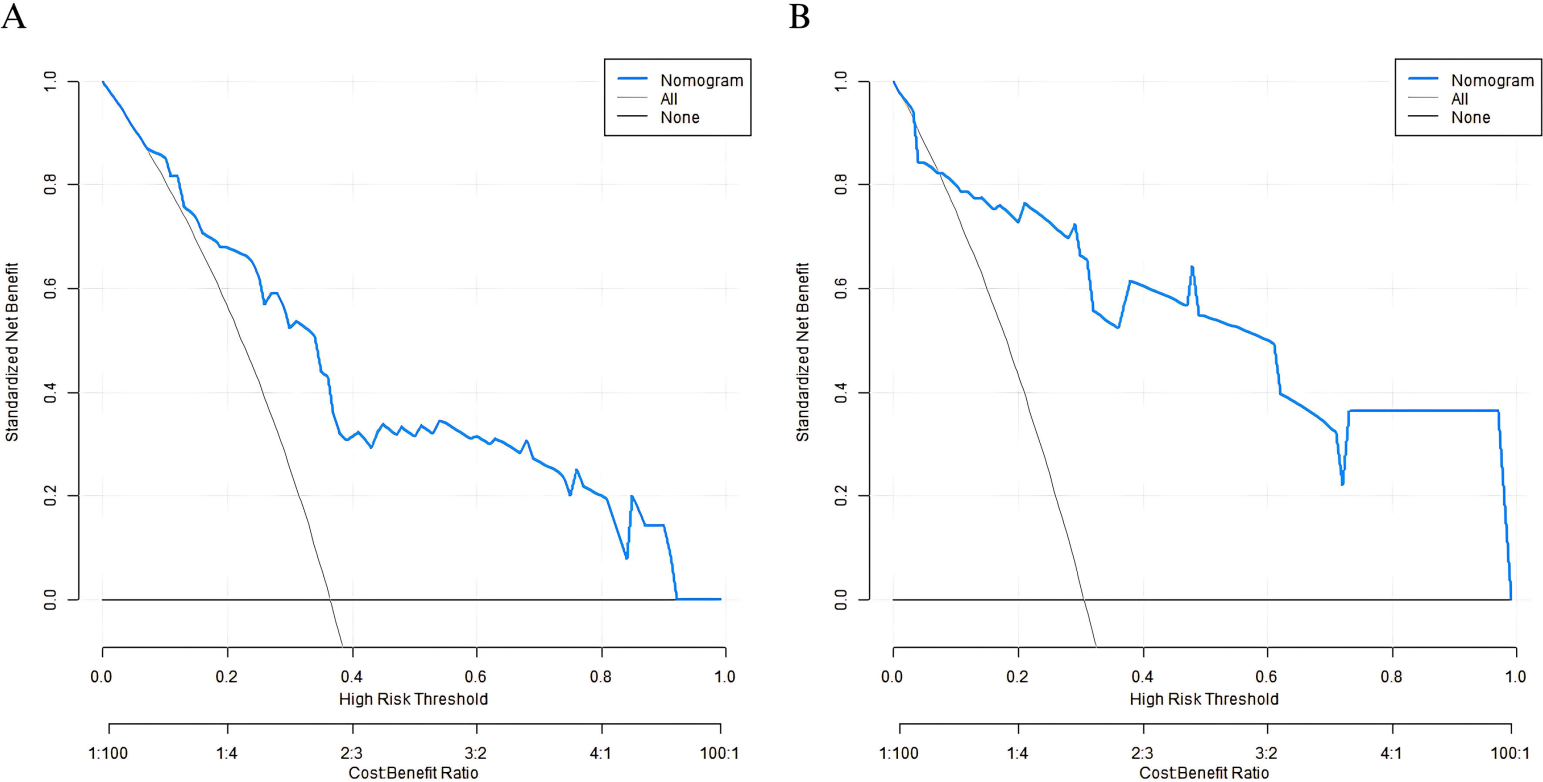
Decision curves in the training set **(A)** and the validation set **(B)**.

Analysis of decision curve showed that when the threshold probability was between 0.08 and 0.85, the decision to apply the Nomogram model constructed in this study to predict the clinical outcome of elderly patients with gastric cancer after surgery had more clinical benefits than the decision that all patients would have adverse clinical outcomes or all patients would not have adverse clinical outcomes before surgery (**Figure 4**).

## 4 Discussion

In this study, we constructed and validated a Nomogram prediction model for clinical outcome of elderly patients with gastric cancer after surgery based on multiple factors, including NLR, BMI, tumor size, lymph node metastasis, CEA and age. This result is of great significance for prognostic evaluation and clinical management of elderly patients with gastric cancer. The developed nomogram provides clinicians with a practical tool for individualized risk assessment. To operationalize this model: (1) For each predictor (x1-x6), locate the patient’s value on the corresponding axis and draw a vertical line to the ‘Points’ axis to determine the partial score. (2) Sum all partial scores to obtain the Total Points. (3) Locate the Total Points on the bottom axis and draw a vertical line to the ‘Disease Risk’ axis to read the predicted probability. For illustration, consider a patient with the following profile: NLR (x1)=3 (50 points), BMI (x2)=23 kg/m² (40 points), Tumor size (x3)=1.8 cm (20 points), Lymph node metastasis (x4)=0 (25points), CEA (x5)=5.7 ng/mL (45 points) and Age (x6)=74 years (40 points). The cumulative score of 220 points corresponds to an estimated 50% probability of poor clinical outcomes. This threshold-based stratification enables clinicians to identify high-risk patients who may benefit from enhanced surveillance or early intervention, and optimize resource allocation while maintaining prognostic accuracy.

Based on the nomogram’s total point distribution (0–240 points), we established three clinically actionable risk categories: low-risk (≤155 points, disease risk <30%), intermediate-risk (155–175 points, 30–60% risk), and high-risk (>175 points, >60% risk). Clinically, this stratification guides management: low-risk (standard follow-up), intermediate-risk (enhanced surveillance), and high-risk (adjuvant therapy consideration). The thresholds balance simplicity with individualized accuracy, aligning with the nomogram’s predictive power.

The nomogram’s visual design and manual scoring system enable rapid risk assessment (<5 minutes) during bedside evaluations, supporting immediate clinical decisions (e.g., adjuvant therapy planning or intensified follow-up for high-risk cases). For streamlined workflows, electronic health record (EHR) systems allows automatic extraction of patient data (age, NLR, BMI, tumor size, lymph node metastasis status, CEA) and generates real-time risk predictions, ensuring scalability across healthcare settings. Crucially, the model is exclusively applicable to elderly gastric cancer patients meeting this study’s inclusion criteria: those undergoing radical surgery without other malignancies or severe organ dysfunction (e.g., severe heart/liver/kidney failure), with complete preoperative NLR and tumor-specific data (including verified tumor size, lymph node metastasis status, and CEA levels). It is not recommended for patients with incomplete clinical data (e.g., missing preoperative NLR) or those receiving non-surgical treatments (e.g., palliative care for advanced unresectable tumors), as predictive performance remains unvalidated in these populations.

NLR, as an indicator reflecting the inflammation and immune state of the body, has been shown to have a significant impact on the clinical outcome of postoperative gastric cancer in the elderly in this study. The occurrence and development of tumors are closely related to the imbalance of inflammatory microenvironment and immune function (8). The results of a previous meta-analysis provided strong or highly suggestive evidence supporting the association between NLR and cancer prognosis (8). Previous study has suggested that NLR might be particularly pertinent to the prognosis of GC patients. In conclusion, the inflammatory markers NLR, PLR, and LMR serve as effective biomarkers for prognostic assessment in GC patients (9). The association between elevated NLR and poor postoperative prognosis may be attributed to its role in shaping an immunosuppressive tumor microenvironment (10). The increased NLR implies enhanced inflammatory response and immunosuppression of the body, which may create favorable conditions for the proliferation, invasion and metastasis of tumor cells (11). In elderly patients with gastric cancer, the immune function is inherently relatively weak due to body function decline. The increased NLR further aggravates the immune disorder, which is not conducive to postoperative recovery and increases the risk of adverse clinical outcomes (12).

BMI is also an important factor affecting the clinical outcome of postoperative gastric cancer in the elderly. Obesity (higher BMI) has been associated with poor prognosis in many cancers, as well as in elderly patients with gastric cancer (13). On the one hand, obese patients have excessive adipose tissue, which can secrete a variety of cytokines and hormones, such as leptin and adiponectin, which regulate the growth, proliferation and invasion of tumor cells. Leptin can activate signaling pathways in tumor cells, promote cell proliferation and resist apoptosis. Abnormal adiponectin levels may affect the metabolism and immune escape of tumor cells. On the other hand, obesity increases the difficulty of surgery and the risk of postoperative complications, such as wound infection and pulmonary infection, and thus affects the patient’s rehabilitation process, leading to adverse clinical outcomes (14).

The tumor size directly reflects the tumor load degree. Larger tumors tend to mean a longer course of disease, greater invasiveness, greater susceptibility to invasion of surrounding tissues and organs, and greater likelihood of distant metastasis (15). For elderly patients with gastric cancer, the body has a poor tolerance to tumors. The larger the tumor, the more serious the consumption of the body. The greater the difficulty and risk of surgical resection. Even with surgical treatment, the potential for residual tumor cells is relatively high, which undoubtedly increases the risk of postoperative recurrence and adverse clinical outcomes (16). In clinical practice, for elderly patients with large tumors, the feasibility of surgery should be carefully evaluated and a comprehensive treatment plan should be formulated to improve the prognosis.

Lymph node metastasis is an important pathway for tumor metastasis and also one of the key factors affecting the prognosis of patients with gastric cancer (17). Once lymph node metastasis occurs, the tumor cells have crossed the barrier of local tissue and entered the lymphatic circulation system, which indicates a higher risk of recurrence and a worse prognosis. Elderly patients with gastric cancer have a weak resistance to tumor metastasis due to decreased body function, and it is more difficult to control the progression of the disease through autoimmune and therapeutic means after lymph node metastasis. In addition, the number and location of lymph node metastasis are also closely related to the prognosis. the more metastatic lymph nodes and the farther the location, the shorter the survival time of patients and the higher the incidence of adverse clinical outcomes (18). The lymph node metastasis should be fully considered in the development of treatment plan, and postoperative adjuvant treatment should be strengthened to reduce the risk of recurrence.

As a common tumor marker, CEA has important significance in the diagnosis and treatment of gastric cancer (19). When gastric cancer occurs, tumor cells secrete CEA, resulting in an increase in its level in the blood. High CEA levels are often associated with high tumor invasiveness and poor prognosis. In this study, CEA was also an independent risk factor for poor clinical outcomes in elderly patients after gastric cancer surgery (20). CEA can not only serve as a marker for tumor presence but also reflect tumor biological behavior. Elevated CEA levels may indicate enhanced proliferative, invasive, and metastatic capacities of tumor cells, and may also reflect CEA-mediated immunosuppression in the tumor microenvironment—such as impaired T-cell cytotoxicity or increased regulatory T-cell infiltration, which weakens the ability of body to clear residual tumor cells. Monitoring changes in CEA levels can help to evaluate the efficacy of treatment and predict the risk of recurrence, which is of great value for dynamic management of elderly patients with gastric cancer.

Age is a factor that cannot be ignored in elderly patients with gastric cancer. With the growth of age, the functions of various organs of the body of elderly patients gradually decline, and their tolerance to surgery and treatment is significantly reduced (21). Elderly patients are often accompanied by a variety of chronic diseases, such as hypertension, diabetes, coronary heart disease, and so on. These complications will increase the risk of surgery and the incidence of postoperative complications. At the same time, the elderly will also lead to decreased immune function of the body, making it easier for tumor cells to escape from the immune monitoring and attack of the body (22). During the treatment, the elderly patients may not be able to tolerate the standard dose of radiotherapy and chemotherapy, thus affecting the therapeutic effect and leading to an increased risk of adverse clinical outcomes. Therefore, when formulating treatment plan, we need to fully consider the age and physical condition of elderly patients and carry out individualized treatment.

Although the Nomogram prediction model constructed in this study showed good calibration degree and prediction performance in the training set and validation set, it still had certain limitations. First, all the samples in this study were from the same hospital, and there might be a certain selection bias. Patients in different regions and hospitals have differences in race, living habits, and medical level, which may affect the universality of the model. Second, while our sample size met minimum requirements for predictive modeling, the single-center derivation may affect generalizability. Formal power calculations were not performed for outcome prediction; future multicenter studies with pre-specified sample sizes based on expected event rates will be valuable to confirm our findings. Third, no external validation has been performed, making the applicability of the model in other clinical settings unclear. However, the sample size of this study was relatively limited, and external validation required a large amount of time, manpower and material resources, and involved a complex process of multi-center collaboration, which faced great difficulties in the development of this study, which was the main reason for the lack of external validation. Large-scale, multi-center external validation is a critical step for enhancing and demonstrating the generalizability and clinical utility of machine learning models. External validation using multi-center cohorts with diverse demographic and clinical characteristics is essential to confirm our findings. In addition, the lack of systematic data on perioperative variables (e.g., surgical approach (laparoscopic vs. open), extent of lymphadenectomy, intraoperative blood loss, and anesthesia techniques) may introduce unmeasured confounding. Future multicenter studies with standardized perioperative data collection are needed to validate and refine our model by incorporating these clinically relevant parameters.

In this study, we successfully constructed a Nomogram prediction model for the clinical outcome of postoperative gastric cancer in the elderly based on multiple factors such as NLR, BMI, tumor size, lymph node metastasis, CEA, and age. The model has good calibration degree and prediction performance in the training set and the validation set, and has certain clinical application value. It can provide a reference for clinicians to predict the clinical outcome of elderly patients with gastric cancer after surgery, and help to develop personalized treatment options. However, since no external validation is conducted in this study, the universality of the model needs to be further verified. In future studies, we should expand the sample size and carry out multi-center research to further improve and verify the model, so as to better serve the clinical diagnosis and treatment of elderly patients with gastric cancer and improve the survival rate and quality of life of patients.

## Data Availability

The raw data supporting the conclusions of this article will be made available by the authors, without undue reservation.
